# Comparison of Injury Severity Score, New Injury Severity Score, Revised Trauma Score and Trauma and Injury Severity Score for Mortality Prediction in Elderly Trauma Patients

**DOI:** 10.5005/jp-journals-10071-23120

**Published:** 2019-02

**Authors:** Rameshbabu Homanna Javali, Krishnamoorthy, Akkamahadevi Patil, Madhu Srinivasarangan, Suraj, Sriharsha

**Affiliations:** 1-6 Department of Emergency Medicine, JSS Medical College, JSS Academy of Higher Education and Research, Mysuru, Karnataka, India

**Keywords:** Elderly, Injury severity score, Mortality, New injury severity score, Revised trauma score, Trauma and injury severity score, Trauma

## Abstract

**Objectives:**

This study tests the accuracy of the Injury Severity Score (ISS), New Injury Severity Score (NISS), Revised Trauma Score (RTS) and Trauma and Injury Severity Score (TRISS) in prediction of mortality in cases of geriatric trauma.

**Design:**

Prospective observational study

**Materials and methods:**

This was a prospective observational study on two hundred elderly trauma patients who were admitted to JSS Hospital, Mysuru over a consecutive period of 18 months between December 2016 to May 2018. On the day of admission, data were collected from each patient to compute the ISS, NISS, RTS, and TRISS.

**Results:**

Mean age of patients was 66.35 years. Most common mechanism of injury was road traffic accident (94.0%) with mortality of 17.0%. The predictive accuracies of the ISS, NISS, RTS and the TRISS were compared using receiver operator characteristic (ROC) curves for the prediction of mortality. Best cutoff points for predicting mortality in elderly trauma patient using TRISS system was a score of 91.6 (sensitivity 97%, specificity of 88%, area under ROC curve 0.972), similarly cutoff point under the NISS was score of 17(91%, 93%, 0.970); for ISS best cutoff point was at 15(91%, 89%, 0.963) and for RTS it was 7.108(97%,80%,0.947). There were statistical differences among ISS, NISS, RTS and TRISS in terms of area under the ROC curve (*p* <0.0001).

**Conclusion:**

TRISS was the strongest predictor of mortality in elderly trauma patients when compared to the ISS, NISS and RTS.

**How to cite this article:**

Javali RH, Krishnamoorthy *et al*. Comparison of Injury Severity Score, New Injury Severity Score, Revised Trauma Score and Trauma and Injury Severity Score for Mortality Prediction in Elderly Trauma Patients. Indian J of Crit Care Med 2019;23(2):73-77.

## INTRODUCTION

**T**rauma is one of the leading causes of mortality and morbidity worldwide^1^. Injuries in the elderly are rapidly becoming a major public health concern^2^.

An appropriate scoring system to predict the mortality and morbidity in elderly is necessary because the elderly population has continued to grow and will continue to represent an increasing proportion of patients in our trauma bays. Additionally, elderly trauma patients have an increased morbidity and mortality for a given severity of injury, a decreased 5-year survival compared to their uninjured counterparts, as well as an increase in intensive care unit (ICU) stays and longer hospital length of stay^3–6^.

Nonetheless, aggressive treatment for elderly trauma patients is paramount, as patients who survive such events often return to independent living^7^.

Trauma scores were introduced more than 30 years ago for assigning numerical values to anatomical lesions and physiological changes after an injury. Physiological scores describe changes due to a trauma and translated by changes in vital signs and consciousness. Anatomical scores describe all the injuries recorded by clinical examination, imaging, surgery or autopsy. If physiological scores are used at first contact with the patient (for triage) and then repeated to monitor patient progress, anatomical scores are used after the diagnosis is complete, generally after patient discharge or postmortem. They are used to stratify trauma patients and to measure lesion severity. Scores that include both anatomical and physiological criteria (mixed scores) are useful for patient prognosis^8^.

### Anatomical Scoring Systems

- Abbreviated injury scale (AIS)
- Injury severity score (ISS)
- New injury severity score (NISS)
- Organ injury scale (OIS)
- Anatomic profile
- International Classification of Diseases (ICD-9) Injury Severity Score (ICISS)

### Physiological Scoring Systems

- Revised trauma score
- Glasgow coma score
- APACHE scoring (Acute physiology and chronic health evaluation (APACHE I, II, III)

### Combination of Anatomic and Physiological Scoring Systems

- Trauma and injury severity scores (TRISS)
- A severity characterization of trauma (ASCOT)

### Injury Severity Score (ISS)

The injury severity score (ISS) is an anatomical scoring system that provides an overall score for patients with multiple injuries. Each injury is assigned an abbreviated injury scale (AIS) score and is allocated to one of six body regions. The highest AIS score in each body region is used. The three most severely injured body regions have their score squared and added together to produce the ISS score^9^.

**Graph 1:**
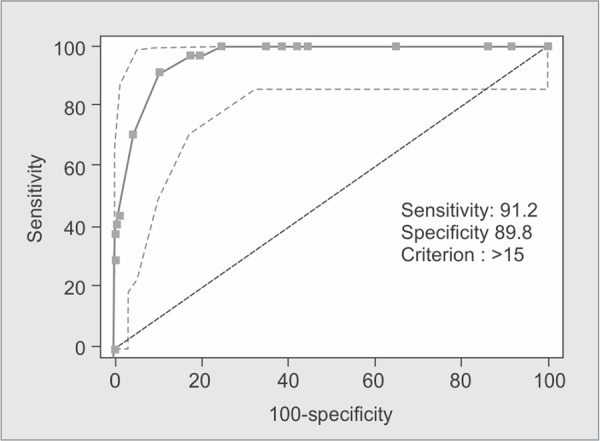
ROC curve showing validity of ISS score in predicting outcome (i.e. mortality)

The ISS score takes values from 0 to 75. If an injury is assigned an AIS of 6 (Unsurvivable injury), the ISS score is automatically assigned to 75. The ISS score is virtually the only anatomical scoring system in use and correlates linearly with mortality, morbidity, hospital stay and other measures of severity (Graph 1).

Major trauma is considered when ISS> 15.

Bolorunduro *etal.* categorised and validated the ISS as follows^10^:

- <9 = Mild
- 9 – 15 =Moderate
- 16–24 = Severe
- >/=25 = Profound

The most important drawback of the ISS is that it only considers one injury in each body region. This leads to injuries being overlooked and to less severe injuries occurring in other body regions being included in the calculation over more serious ones in the same body region^11^.

### New Injury Severity Score (NISS)

**Graph 2:**
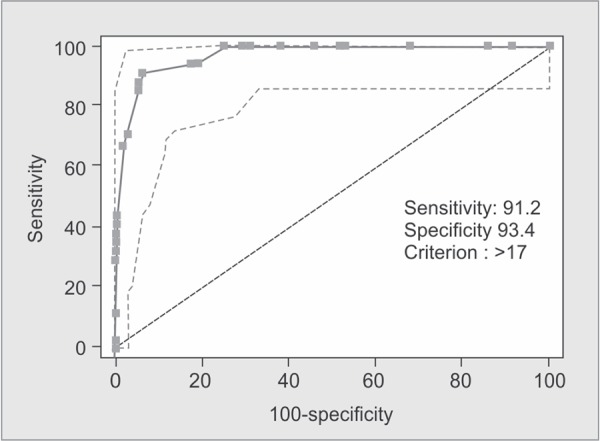
ROC curve showing validity of NISS score in predicting outcome (i.e. mortality)

A simple modification to the ISS, the new injury severity score (NISS), was designed by Osler *et al.* in 1997 to counter this problem^12^. The NISS is simply the sum of squares of the three most severe injuries, regardless of body region injured. Therefore, the NISS will be equal to or higher than the ISS (Graph 2).

### Revised Trauma Score (RTS)

**Graph 3:**
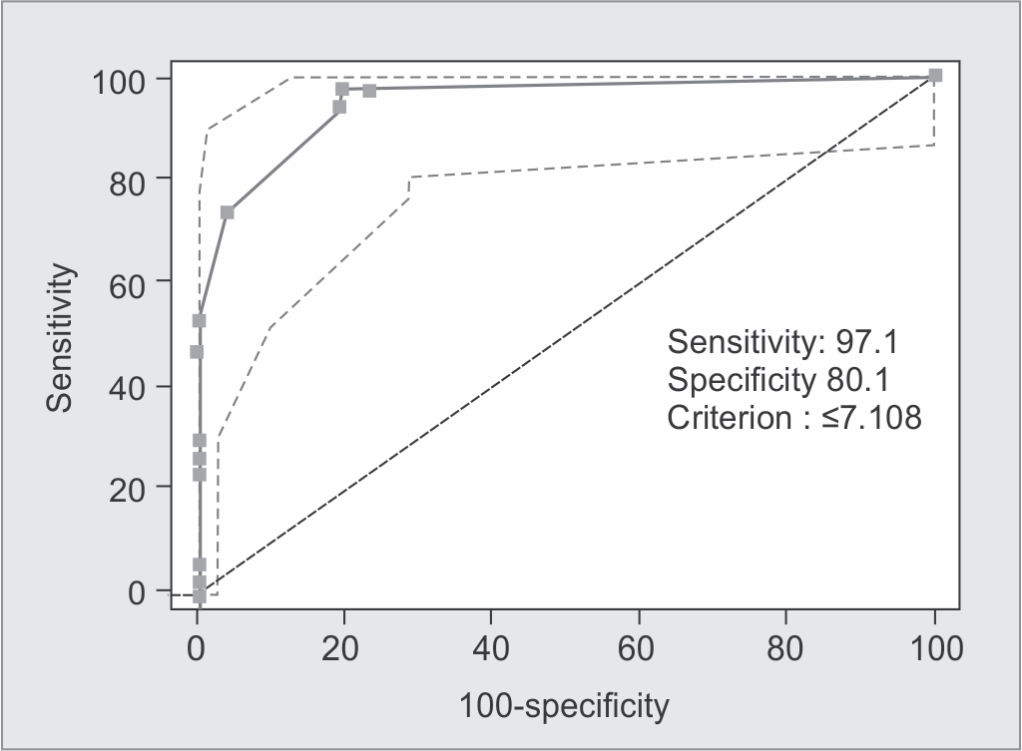
ROC curve showing validity of RTS score in predicting outcome (i.e. mortality)

The revised trauma score is a physiological scoring system, with high interrater reliability and demonstrated accuracy in predicting death. It is scored from the first set of data obtained on the patient and consists of glasgow coma scale, systolic blood pressure and respiratory rate (Graph 3)^13^.

**RTS = 0.9368 GCS + 0.7326 SBP + 0.2908 RR**

RTS score coding is shown in Table 1. Values for the RTS are in the range 0-7.8408. The RTS is heavily weighted towards the Glasgow coma scale to compensate for major head injury without multisystem injury or major physiological changes. A threshold of RTS <4 has been proposed to identify those patients who should be treated in a trauma centre, although this value may be somewhat low.

### Trauma and Injury Severity Scores (TRISS)

TRISS determines the probability of survival (Ps) of a patient from the ISS and RTS using the following formulae:

Ps= 1/ (1 + e^-b^)
Where ‘b’ is calculated from:
b = b0 + b1 (RTS) + b2 (ISS) + b3 (Age Index)

**Graph 4:**
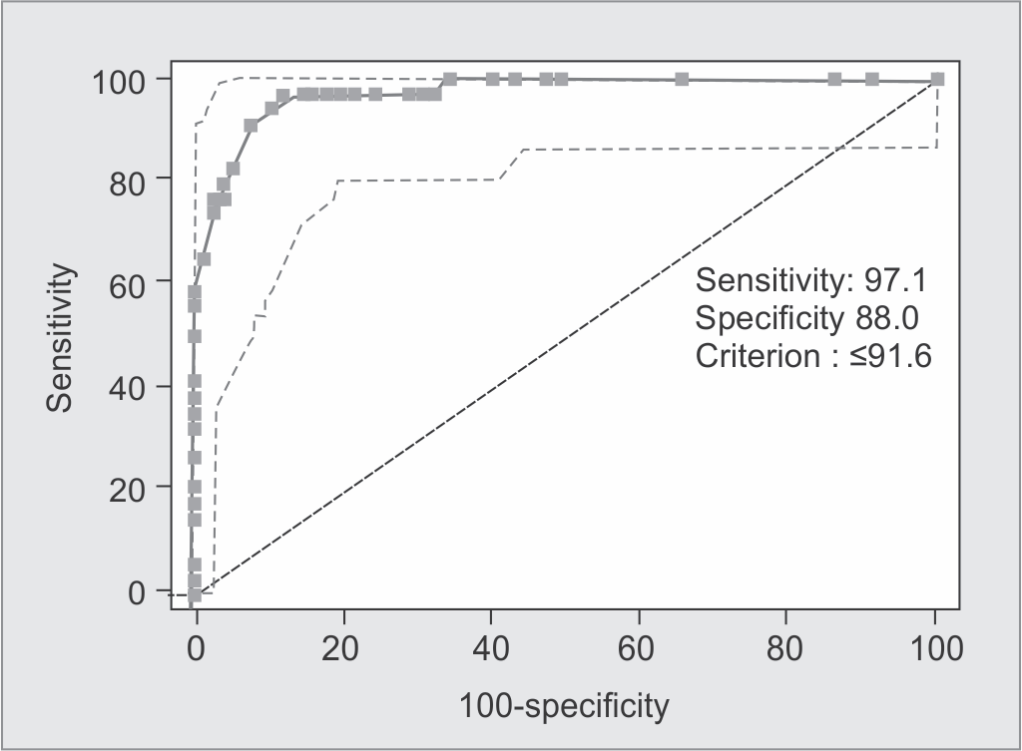
ROC curve showing validity of TRISS score in predicting outcome (i.e. mortality)

The coefficients b0 - b3 are derived from multiple regression analysis of the Major Trauma Outcome Study (MTOS) database. Age Index is 0 if the patient is below 54 years of age or 1 if 55 years and over. b0 - b3 are coefficients which are different for blunt and penetrating trauma. If the patient is less aged than 15 years, the blunt coefficients are used regardless of mechanism (Graph 4).

**Table 1:** RTS score coding

*Glasgow coma scale (GCS)*	*Systolic blood pressure (SBP)*	*Respiratory rate (RR)*	*Coded value*
13-15	>89	10-29	4
9-12	76-89	>29	3
6-8	50-75	6-9	2
4-5	1-49	1-5	1
3	0	0	0

**Table:** 

	*Blunt*	*Penetrating*
b0	-0.4499	-2.5355
b1	0.8085	0.9934
b2	-0.0835	-0.0651
b3	-1.7430	-1.1360

The TRISS calculator determines the probability of survival from the ISS, RTS and patient's age. ISS and RTS scores can be given independently or calculated from their base parameters^14^.

TRISS uses combination of both anatomic and physiologic scoring systems and gives a more accurate probability of survival.

## MATERIALS AND METHODS

Our study was a prospective observational study carried out over a period of 18 months (December 2016 - June 2018) on elderly victims of trauma presenting to the Department of Emergency Medicine. Patients were selected after taking into account the inclusion and exclusion criteria (Flowchart 1).

### Inclusion Criteria

- Age more than 60 years.
- History and clinical evidence of trauma.

**Flowchart 1:**
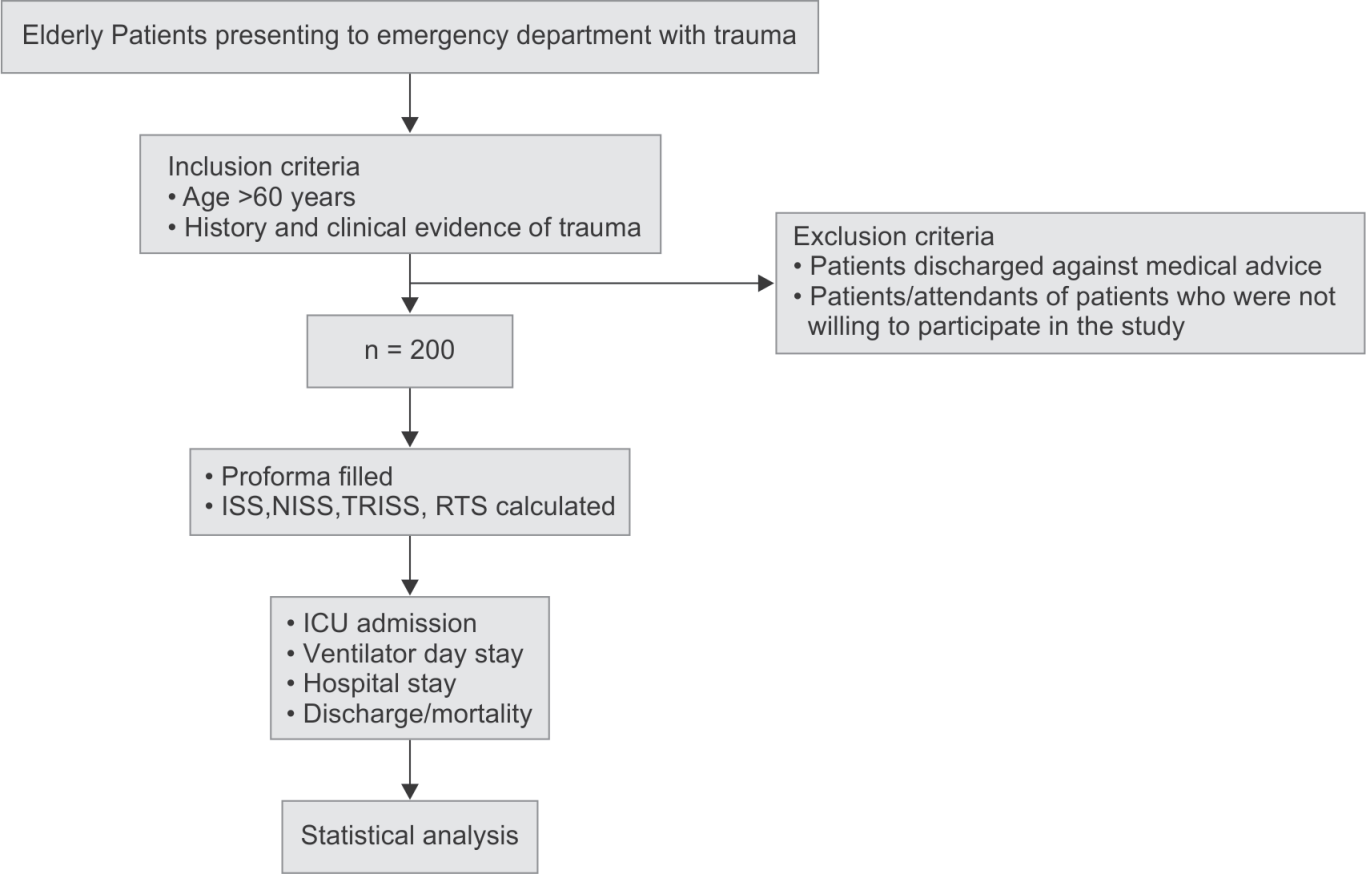
Methodology of our study-depicting research

#### Exclusion Criteria

- Patients discharged against medical advice.
- Patients/ Attendants of patients who were not willing to participate in the study.

### DATA ANALYSIS

The sensitivity, specificity and correct prediction of outcome for each cutoff point were calculated for ISS, NISS, RTS and TRISS. The best cutoff point in each scoring system is determined when the point yields the best specificity and sensitivity in the two-by-two Table. The best Youden index also determines the best cutoff point. The Youden index was used to compare the proportion of cases correctly classified. The higher the Youden index, the more accurate is the prediction (higher true positive and true negatives and fewer false positive and false negatives) at the cutoff point^15^. Descriptive statistics were expressed as mean ± one standard deviation unless otherwise stated. A receiver operating characteristic (ROC) curve depicts the relation between true-positive and false-positive for each scoring system. This method compares scores without fixing arbitrary cutoff points. The area under ROC curve was calculated and evaluated. AUROC represents that a randomly chosen diseased subject is more correctly rated or ranked than a randomly chosen non-diseased subject^16^.

A value of 0.5 under the ROC curve indicates that the variable performs no better than chance and a value of 1.0 indicates perfect discrimination. A larger area under the ROC curve represents more reliability and good discrimination of the scoring system^17^.

### RESULTS

Two hundred trauma patients aged more than 60 years were included in our study with a mean age of 66.35 ± 6.865 years. Of the total, 74% were men (n= 148) and 26% were women (n=52). Most common mechanism of trauma was road traffic accident (94%), followed by fall (5.5%). All cases were blunt trauma. 166 patients (83%) recovered after treatment in hospital and 34 patients (17%) died (Table 2).

**Table 2:** Epidemiological findings

*Variables*		*Cases*	*%*
age	60-70 years	160	80.0
	71-80 years	34	17.0
	>80 years	6	3.0
Sex	Female	52	26.0
	Male	148	74.0
	Assault	1	0.5
Mode of injury	Fall	11	5.5
	RTA	188	94.0
Nature of Injury	Blunt	200	100.0
	Penetrating	0	0
Outcome	Discharged	166	83.0
	Mortality	34	17.0
	Head-neck	102	51
	Face	81	40.5
Region of trauma	Thorax	17	8.5
	Abdomen	4	2
	Extremity	84	42
	External	110	55

Among those who were discharged, mean± SD of ISS, NISS, RTS and TRISS scores were 7.52±5.03, 8.80±6.19, 7.60±0.48 and 95.49±4.41 respectively. Among those who were expired, mean ± SD of ISS, NISS, RTS and TRISS scores were 20.85 ±5.00, 27.65 ±7.49, 5.43±1.29 and 58.48 ±25.58 respectively (Table 3).

In our study mean trauma scores of ISS and NISS were significantly higher in expired subjects than in Living subjects, RTS and TRISS were significantly lower in expired subjects than in Living subjects.

The predictive accuracies of the ISS, NISS, RTS and the TRISS were compared using receiver operator characteristic (ROC) curves for the prediction of mortality. Best cutoff points for predicting mortality in elderly trauma patients in ISS, NISS, RTS and TRISS systems were 15, 17, 7.108, 91.6 with sensitivity of 91%, 91%, 97%, 97% and specificity of 89%, 93%, 80%, 87%, respectively.

The area under ROC curve was 0.963, 0.970, 0.947, 0.972 in the ISS, NISS, RTS and TRISS respectively. The Youden index had best cut-off points at 0.80, 0.84, 0.77 and 0.85 for ISS, NISS, RTS and TRISS respectively (Table 4).

### DISCUSSION

Most of elderly trauma patients in the present study were men. Some studies have shown similar results^18,19^. Mean age of our patients was 66.35 years with age range of 60 to 95 years. This is lesser than other studies^20,21^. The present study indicated that the most common mechanisms leading to trauma in the elderly were motor vehicle accidents, followed by falls. A study by Aydin *et al*. also revealed that most common mechanism leading to trauma was motor vehicle accident^22^. Most frequently injured organ was extremity followed by head and neck. Aydin *et al.* study also reported that the most common injury sites were head and extremities^22^. In this study, 17% of elderly trauma patients died. Yousefzadeh-Chabok *etal.* study reported mortality rate of 13.9%^23^.

In the present study, mean plus standard deviation of ISS for non-survivors was 20.85±5.00 and 7.52±5.03 for survivors; mean plus standard deviation of NISS for non-survivors was 27.65±7.49 and 8.80±6.19 for survivors, mean plus standard deviation of RTS for non-survivors was 5.43±1.29 and 7.60±0.48 for survivors. Mean plus standard deviation of TRISS for non-survivors was 58.48±25.58 and was 95.49± 4.41 for survivors.

In a study by Orhon *et al.*, mean plus standard deviation of ISS for non-survivors was 24.37 ± 12.85 and 5.78 ± 6.71 for survivors;; mean plus standard deviation of NISS for former was 27.62 ± 12.85 and 6.92 ± 8.13, mean plus standard deviation of RTS for former was 5.62 ± 1.31, and 7.75 ± 0.46 for latter group. Mean plus standard deviation of TRISS for non-survivors was 72.80 ± 19.35 and was 98.34±6.58 for survivors^24^.

In a study by Yousefzadeh-Chabok *et al*., mean plus standard deviation of ISS for non-survivors was 15.95±10.46 and 7.31±6.22 for survivors; mean plus standard deviation of RTS for former was 5.65±1.82, and 7.79±0.27 for latter group. Mean plus standard deviation of TRISS for non-survivors was 1.04±1.49 and was 3.49±0.6 for survivors^23^.

**Table 3:** Mean ISS, NISS, RTS, and TRISS of elderly survivors and non-survivors

	*Non-survivors (n = 34)*	*Survivors (n = 166)*	
*Scores*	*Mean ± SD*	*Mean ± SD*	*p value*
ISS	20.85 ± 5.00	7.52 ± 5.03	<0.001*
NISS	27.65 ± 7.49	8.80 ± 6.19	<0.001*
RTS	5.43 ± 1.29	7.60 ± 0.48	<0.001*
TRISS	58.48 ± 25.58	95.49 ± 4.41	<0.001*

**Table 4:** Comparison of the assessment scores in predicting outcome

*Scores*	*Cutoff*	*Sensitivity*	*Specificity*	*PPV*	*NPV*	*AUC*	*Youden index*	*p value*
ISS	>15	91.18%	89.76%	64.6%	98.0%	0.963	0.8094	<0.0001
NISS	>17	91.18%	93.37%	73.8%	98.1%	0.970	0.8455	<0.0001
RTS	≤7.108	97.06%	80.12%	50%	99.3%	0.947	0.7718	<0.0001
TRISS	≤91.6	97.06%	87.95%	62.3%	99.3%	0.972	0.8501	<0.0001

Our results indicated that ISS and NISS value for survivors is significantly lower than for non-survivors. RTS and TRISS value for survivors were higher than non-survivors. This difference was statistically significant.

In the present study, area under ROC curve using ISS, NISS, RTS, and TRISS for predicting death was 0.963, 0.970, 0.947 and 0.972 respectively; all of these scores were statistically significant in terms of mortality prediction.

In Aydin *et al.* study, area under ROC curve using ISS, NISS and TRISS for predicting death was 0.907, 0.914 and 0.934, respectively^22^. In Yousefzadeh-Chabok *et al.* study, area under ROC curve using ISS, RTS and TRISS for predicting death was 0.76, 0.87 and 0.94, respectively^23^.

In a study conducted by Mitchell *et al*. in Canada published in 2007, it was reported that scoring systems including TRISS had a good ability to predict the prognosis of patients with trauma^25^. In a study conducted in India, Hariharan *et al*. concluded that using TRISS system to predict morbidity and mortality after fall in the elderly can play an important role in treatment planning^26^.

According to logistic regression model used in our study, NISS and TRISS were strong predictors of mortality in elderly trauma patients.

#### Drawbacks of Our Study

- The sample size is relatively small.
- This is a single centre study

### CONCLUSION

Our study findings suggest that utility and applicability of injury severity scoring systems in elderly trauma patients using ISS, NISS, RTS, and TRISS scores can better help the emergency physicians in predicting the prognosis. However, TRISS has maximum prediction in outcome when compared with the other scores.
